# Tumor deposits in colorectal cancer: Refining their definition in the TNM system

**DOI:** 10.1002/ags3.12652

**Published:** 2023-01-12

**Authors:** Hideki Ueno, Iris D. Nagtegaal, Philip Quirke, Kenichi Sugihara, Yoichi Ajioka

**Affiliations:** ^1^ Department of Surgery National Defense Medical College Saitama Japan; ^2^ Department of Pathology Radboud University Medical Centre Nijmegen The Netherlands; ^3^ Division of Pathology and Data Analytics University of Leeds, St James's University Hospital Leeds UK; ^4^ Department of Surgical Oncology Tokyo Medical and Dental University, Graduate School of Medical and Dental Sciences Tokyo Japan; ^5^ Division of Molecular and Diagnostic Pathology Niigata University Graduate School of Medical and Dental Sciences Niigata Japan

**Keywords:** Extramural cancer deposits without lymph node structure (EX), Lymph node metastasis, Tumor deposits (TDs), tumor stage, Tumor‐node‐metastasis (TNM) system

## Abstract

Tumor deposits (TDs) are discontinuous tumor spread in the mesocolon/mesorectum which is found in approximately 20% of colorectal cancer (CRC) and negatively affects survival. We have a history of repeated revisions on TD definition and categorization in the tumor‐node‐metastasis (TNM) system leading to stage migration. Since 1997, TDs have been categorized as T or N factors depending on their size (TNM5) or contour (TNM6). In 2009, TNM7 provided the category of N1c for TDs in a case without positive lymph nodes (LNs), which is also used in TNM8. However, increasing evidence suggests that these revisions are suboptimal and only “partially” successful. Specifically, the N1c rule is certainly useful for oncologists who are having difficulty with TDs in a case with no positive LNs. However, it has failed to maximize the value of the TNM system because of the underused prognostic information of individual TDs. Recently, the potential value of an alternative staging method has been highlighted in several studies using the “counting method.” For this method, all nodular type TDs are individually counted together with positive LNs to derive the final pN, yielding a prognostic and diagnostic value that is superior to existing TNM systems. The TNM system has long stuck to the origin of TDs in providing its categorization, but it is time to make way for alternative options and initiate an international discussion on optimal treatment of TDs in tumor staging; otherwise, a proportion of patients end up missing an opportunity to receive the optimal adjuvant treatment.

## Graphical abstract

This article reviewed a history of repeated revisions on tumor deposit (TD) definition and categorization in the tumor‐node‐metastasis (TNM) system, which may have failed to maximize the value of the TNM system because of the underused prognostic information of individual TDs. Recently, the potential value of an alternative staging method has been highlighted in several studies using the “counting method,” in which all nodular type TDs are individually counted together with positive LNs to derive the final pN. The treatment of TDs should be determined in terms of how it will maximize the prognostic value of TNM classification and its reproducibility, and it is time to initiate an international discussion on optimal treatment of TDs in tumor staging; otherwise, a proportion of patients end up missing an opportunity to receive the optimal adjuvant treatment.

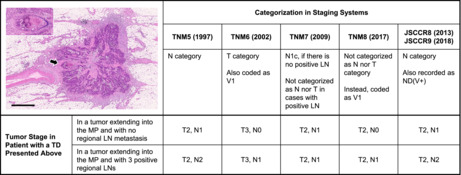

## 1 INTRODUCTION

Tumor staging in conventional pathological investigation is still at the center of clinical reporting on colorectal cancer (CRC) even in the precision medicine era. A recent milestone study by the International Duration of Adjuvant Chemotherapy (IDEA) collaboration highlighted the importance of the sub‐staging of stages II and stage III in determining an adjuvant chemotherapy regimen for individual patients.^1^, ^2^ Currently, T and N stages are unprecedentedly important as they determine the selection of patients for the chemotherapy regimen, thereby determining patient clinical outcomes.^3^

Tumor deposits (TDs) were isolated tumor lesions in the regional lymphatic area other than those in the lymph nodes, which were first noticed in the 1980s by some pathologists, such as Jass and Morson. They described that “the clinical importance of this observation is unknown at present.”^4^ The TDs were first adopted in the Union for International Cancer Control (UICC)'s TNM classification 5th edition in 1997 as a staging factor (Table 1).^5^ Since then, pathological practice for TDs has been revised in every revision of the TNM staging system until its 8th edition (2017). However, uncertainty remained in TD definition and categorization in the tumor staging system. Over time, considerable new data around TDs has accumulated, which should now guide us in the optimization of the TNM staging system.

**TABLE 1:** Definition and categorization of tumor deposits (TDs) in staging systems

Staging system (publication year)	Terminology	Definition	Categorization criteria for tumor staging
TNM5 (1997)	Tumor nodule	A nodule in perirectal or pericolic adipose tissue without histological evidence of a residual lymph node in the nodule.	A tumor nodule of >3 mm in diameter is classified as regional LNM; a tumor nodule up to 3 mm in diameter is classified in the T category as a discontinuous extension, i.e., T3.
TNM6 (2002)	Tumor nodule	A tumor nodule in the pericolic or perirectal adipose tissue without histological evidence of residual lymph node in the nodule.	If the nodule has the form and smooth contour of a lymph node, it is classified in the pN category as a regional LNM; if the nodule has an irregular contour, it should be classified in the T category and also coded as V1 (microscopic venous invasion) or V2, if it was grossly evident, because there is a strong likelihood that it represents venous invasion.
TNM7 (2009)	Tumor deposits (satellites)	Macroscopic or microscopic nests or nodules in the pericolorectal adipose tissue's lymph drainage area of a primary carcinoma without histological evidence of residual lymph node in the nodule.	If tumor deposits are observed with lesions that would otherwise be classified as T1 or T2, then the T classification is not changed, but the nodule(s) is recorded as N1c. If a nodule is considered by the pathologist as a totally replaced lymph node (generally having a smooth contour), it should be recorded as a positive lymph node and not as a satellite, and each nodule should be separately counted as a lymph node in the final pN determination.
TNM8 (2017)	Tumor deposits (satellites)	Discrete macroscopic or microscopic nodules of cancer in the pericolorectal adipose tissue's lymph drainage area of a primary carcinoma that are discontinuous from the primary and without histological evidence of residual lymph node or identifiable vascular or neural structure.	If a vessel wall is identifiable on H&E, elastic, or other stains, it should be classified as a venous invasion (V1/2) or lymphatic invasion (L1). Similarly, the lesion should be classified as a perineural invasion (Pn1) if neural structures are identifiable. The presence of tumor deposits does not change the primary tumor T category but changes the node status (N) to pN1c if all regional lymph nodes are negative on pathological examination.
JSCCR8 (2013) JSCCR9 (2018)	Extramural cancer deposits without lymph node structure (EX)	Extramural cancer deposits with no lymph node structure within the regional lymph node area. EX includes localized lesions comprising lymphatic invasion, venous invasion, perineural invasion (vascular/perineural invasion lesions), and other lesions (tumor nodule: [ND]). All tumor deposits located in the extramural fatty tissue are regarded as EX in tumors in which continuous spread is confined within the SM or MP. Tumor deposits located ≥5 mm from the leading edge of the primary tumor are designated as EX for tumors that directly penetrate the MP.	ND is treated as LNM and each ND is separately counted as a lymph node in the final pN determination. Vascular/perineural invasion lesions are treated as T‐factor, thereby changing the final pT determination (i.e., T3) in tumors that would otherwise be classified as T1 or T2. ND with histological evidence of venous invasion or perineural invasion in the nodule is recorded with a symbol of ND(V+) or ND(Pn+) because it represents a strong likelihood of getting a poor prognosis.

Abbreviation: EX, extramural cancer deposit without lymph node structure; H&E, hematoxylin and eosin staining; JSCCR, Japanese Society for Cancer of the Colon and Rectum; LNM, lymph node metastasis; MP, muscularis propria; ND(Pn+), tumor nodule with histological evidence of perineural invasion in the nodule; ND(V+), tumor nodule with histological evidence of venous invasion in the nodule; ND, tumor nodule without histological evidence of residual lymph node structure; SM, submucosal layer.

## 2 PROGNOSTIC IMPACT OF TDS

**FIGURE 1:**
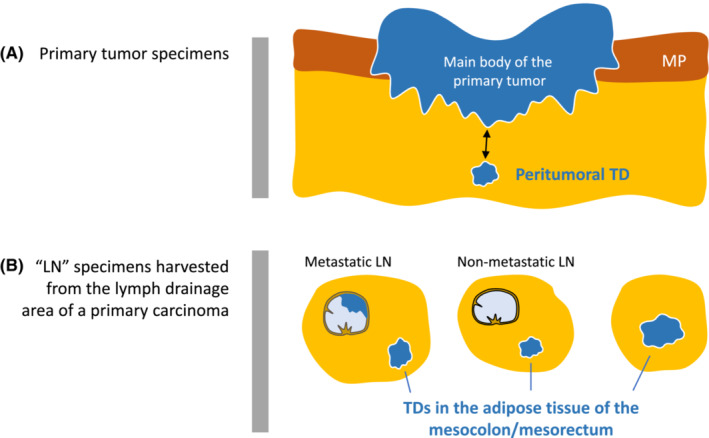
Area of adipose tissue harboring tumor deposit (TD). TDs exist in extramural adipose tissue attached to the bowel wall with the primary tumor (A) and in a lump of adipose tissue postoperatively harvested for pathologic examination of LN metastasis (B). Regarding the length of discontinuity to define peritumoral TD (two‐headed arrow in [A]), a yardstick of 5‐mm discontinuity is used as a criterion for judging a peritumoral TD in the Japanese classification of colorectal, appendiceal, and anal carcinoma (third English edition), i.e., only a deposit discontinuously located at ≥5 mm from the main body of the primary tumor is attributed to the final pathological stage (*see* Figure 5). LN, Lymph node; MP, Muscularis propria; TD, Tumor deposit

Early studies of the prognostic impact of TDs date back to the 1990s. Harrison et al. investigated metastatic tumor nodules in perirectal or pericolic fat with the definition of “discrete aggregates of carcinoma within fibroadipose tissue unassociated with recognizable lymph node (LN) structure and not contiguous with the mural component of invasive carcinoma” and showed that such lesions adversely affected the prognosis in rectal cancer (1994)^6^ and right‐sided colon cancer (1995).^7^ In 1997, Ueno and Mochizuki first reported the actual status of discontinuous cancer‐spread lesions based on the systematic investigation of the lymph drainage area of a primary carcinoma, i.e., not only extramural adipose tissue attached to the bowel but also adipose tissue of the regional lymphatic area that was postoperatively harvested for pathologic examination of LNs (Figure 1).^8^ Their study reported a 26% incidence rate of such lesions in patients with curative resection and 66% in those with non‐curative resection. They categorized the discontinuous lesions into four patterns: scattering, vessel invasion, neural invasion, and nodular type, and all types notably exerted an adverse impact on survival.

Since the first decade of the 2000s, an increasing number of papers has been published on the adverse prognostic impact of TDs in CRC. The meta‐analysis by Nagtegaal et al. revealed an average 22% incidence of TDs (range, 5%–42%) in a total of 10 106 patients in 17 studies, which variously included stages I–IV colon or rectal cancer, depending on the study.^9^ The hazard ratio (HR) on the prognostic magnitude of TDs was 2.2 (1.6–3.0) on disease‐free survival (DFS) (five studies, 1246 patients), 3.3 (2.2–4.7) on disease‐specific survival (five studies, 4446 patients), and 2.9 (2.2–3.8) on overall survival (OS) (three studies, 814 patients) in univariate analyses. Importantly, the inclusion of additional variance did not diminish the significance of the HR for TD even in the multivariable models.^9^

Goldstein and Turner reported that the presence of TDs is an independent poor prognostic factor and insisted that TDs are distinct from LN metastases (LNM) and should not be considered their prognostic equivalent.^10^ Actually, the background of the tumor is different between patients with positive LNM and TD, where TD is more likely to appear in advanced tumors, with a lower 5‐year survival rate in patients positive for TD than LNM.^11^ However, the HR between positive LNM and TD were not statistically different, demonstrating 4.1–4.5 for LNM and 4.0–5.3 for TD, respectively.^11^

## 3 HISTOLOGICAL DEFINITIONS OF TDS

Varying terminology has been used in the literature to describe the lesions associated with TDs, such as metastatic tumor nodules,^6^, ^7^ extra‐bowel skipped cancer infiltration,^8^ mesorectal microfoci,^12^ non‐nodal metastatic foci,^13^ soft tissue implants of tumors,^13^ and extramural discontinuous cancer spread.^11^ Over time, these terms have gradually unified as TDs. However, the definition of TDs has not yet been standardized effectively even in the UICC/American Joint Committee on Cancer (AJCC) staging system, where pathological definition of TDs has been changing at every revision from TNM5 to TNM8 (Table 1). In its latest edition, some uncertainties remained in TD definition, causing a diagnostic disparity of TDs, followed by stage migration in a certain proportion of patients with CRC (Table 2).

**TABLE 2:** Issues to be solved for future international tumor staging systems regarding the definition and categorization of tumor deposits (TDs)

Points at issue in the TNM8	Evidence to date associated with the issue
1. Uncertainty regarding the definition of the “discontinuity” in peritumoral TD diagnosis	There is no international consensus as to the definition of the distance from the advancing edge of the main body of the primary tumor or bowel wall for peritumoral TD. Some criteria have been proposed such as 1) ≥5 mm,^11^, ^26^ 2) ≥1 cm,^23^ and 3) no clear connection.^27^ The “5‐mm” is the only criterion for peritumoral TD that had been predetermined in a multicenter study,^11^, ^26^ and this criterion has been used across Japan since 2013.^19^
2. Uncertainty regarding the appropriateness of isolated intravascular or perineural TDs not being taken into account in tumor staging	Intravascular or perineural TDs have been included in many studies to analyze the prognostic impact of TDs and there is no evidence that the prognostic value of tumor staging is improved by excluding such lesions from staging factors. The prognostic power of the T stage was improved by treating isolated intravascular/perineural deposits as a pT3‐determining factor in tumors otherwise diagnosed as pT1 or pT2.^14^, ^15^
3. Lack of meaningful rationale of distinguishing TDs with identifiable vascular or neural structures from other TDs in tumor staging	None of the studies that investigated the prognostic value of TDs have excluded TDs with identifiable vascular or neural structure from the analyses and there is no evidence that the prognostic value of tumor staging is improved by excluding such TDs from staging factors. Nodular TD accompanied by the finding of venous/perineural invasion in the nodule has a prognostic value that is greater than other types of TDs.^11^, ^15^ Impaired diagnostic reproducibility of tumor staging is inevitable due to interobserver disagreement regarding the judgment for vascular or neural structure in the lesion. Kappa statistic was reportedly 0.61 between nodular TD with venous/perineural invasion and other types of extramural discontinuous lesion.^11^
4. Lack of evidence for the value of the N1c category	A multicenter study showed that TNM7 adopting the N1c category is superior to TNM6 characterized by the contour rule, but not to TNM5 characterized by the size rule in terms of its prognostic power.^26^ Under the N1c rule, the tumor stage does not change according to the number of existing TDs in the resected specimens, but the number of TD has prognostic information.^10^, ^11^, ^36^, ^37^ Impaired diagnostic reproducibility of tumor staging is inevitable due to interobserver disagreement on the distinction between TDs and LNM. Kappa statistic was 0.74 between LNM and TDs,^14^ and 0.38 between “nodal” or “non‐nodal” origin.^18^ An increasing number of studies indicate the value of a modified staging system in which TDs are counted individually same as lymph nodes in the final pN determination (*see* Table 3).

Abbreviations: CI, confidence interval; HR, hazard ratio; TD, tumor deposit.

### 3.1 Uncertainty in the diagnostic criteria for TDs

#### 3.1.1 Intravascular or perineural TDs

**FIGURE 2:**
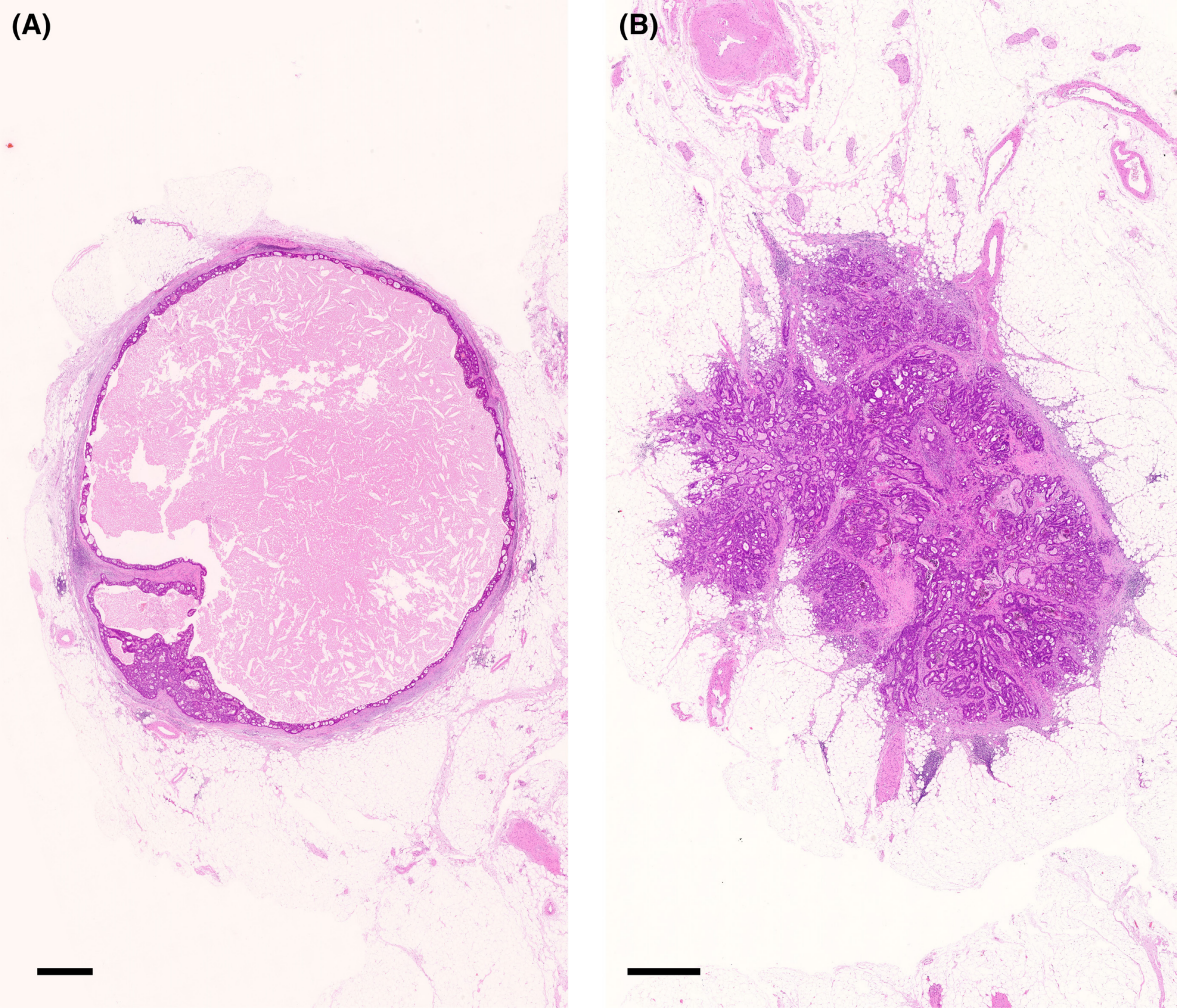
Tumor nodules in the pericolorectal adipose tissue lymph drainage area of a primary carcinoma. In TNM6, tumor nodules without histological evidence of residual lymph node in the nodule are classified in the pN category as a regional lymph node metastasis if the nodule has a smooth contour (A). A nodule with an irregular contour (B) is classified in the T category and also coded as V1 or V2. In TNM7 and TNM8, tumor nodules are no longer treated as a T category. A nodule considered by the pathologists as a totally replaced lymph node is regarded as a positive lymph node, and otherwise, it may change the node status to pN1c depending on some conditions defined differently in TNM7 and TNM8. No specific criteria for a nodule that should be diagnosed as a totally replaced lymph node are being provided other than a short explanatory note that it is “generally having a smooth contour”. (A and B), hematoxylin and eosin staining; *Bar*, 1 mm

**FIGURE 3:**
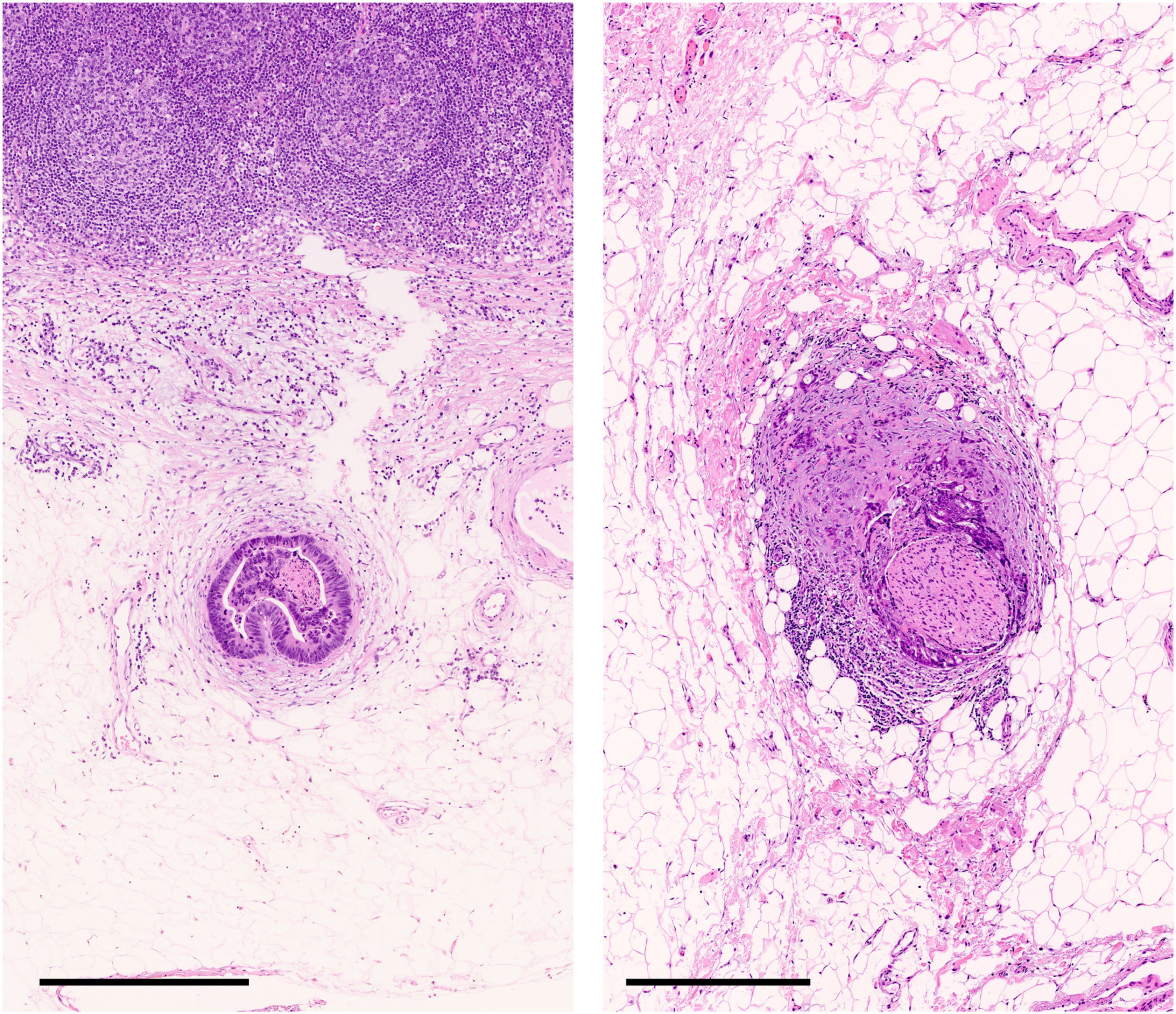
Non‐nodular type tumor deposits in the pericolorectal adipose tissue lymph drainage area of a primary carcinoma. An intravascular tumor deposit located near a non‐metastatic lymph node (A) and a perineural tumor deposit (B) in the regional lymph node area. (A and B), hematoxylin and eosin stain; *Bar*, 500 mm

TDs exist in various forms but can pathologically be categorized into two types, i.e., tumor nodules (ND) (Figure 2) and relatively small deposits of cancer predominantly confined to the vascular (lymphatic or venous vessel) or perineural spaces (intravascular or perineural TDs) (Figure 3). In 1997, TDs first appeared in TNM5 with the term “tumor nodule,” which was also adopted by TNM6. The term “TDs (satellites)” was alternatively used in TNM7, although without specified reason, in which TDs were defined as “macro‐ or microscopic nests or nodules, in the lymph drainage area of a primary carcinoma without histological evidence of residual LN in the nodule.” It is unclear whether TDs under TNM5, TNM6, and TNM7 included non‐nodular TDs, such as intravascular or perineural TDs (Figure 3) that reportedly exist in approximately 3% in the peritumoral adipose tissue located at >5 mm from the primary tumor and 1%–2% in the “LN” specimens (specimens postoperatively harvested for pathological examination of LN metastasis).^14^

A single‐center study focused on the distinction between nodular and intravascular TDs and revealed HRs of 4.7 (3.5–6.2) and 2.5 (1.6–3.8), respectively, and the AIC value for T staging was improved when intravascular TDs were treated as T category.^15^ Additionally, a multicenter study conducted by the Japanese Society for Cancer of the Colon and Rectum (JSCCR), which included two cohorts comprising 1716 patients and 2242 patients, respectively, revealed that the statistic figures representing the performance of tumor staging were improved when intravascular or perineural TDs were treated as a T‐factor.^14^

TNM7 and TNM8 have provided a distinction between perineural and lymphovascular invasions in terms of being treated as a T‐factor and V (venous invasion) or L (lymphatic invasion) classification, respectively.^16^, ^17^ However, the tumor stage, rather than V and L classification, is currently considered mostly as a treatment decision factor in clinical practice; thus, we may better revisit the concept adopted in TNM5 and TNM6 in which prognostic information of all TD types was effectively reflected in either the T or N stage. In Japan, intravascular or perineural TDs are treated differently from nodular TDs in determining the tumor stage. Specifically, intravascular or perineural TDs are regarded as a T‐factor, thereby making a tumor upstage to pT3 if observed in otherwise pT1 or pT2 tumors. This may be comparable to the concept of the TD treatment adopted in TNM5 where a pericolic or perirectal tumor nodule of up to 3 mm in diameter was treated as a T category, i.e., a T3‐determinant factor.^5^

#### 3.1.2 TDs with identifiable vascular or neural structure

**FIGURE 4:**
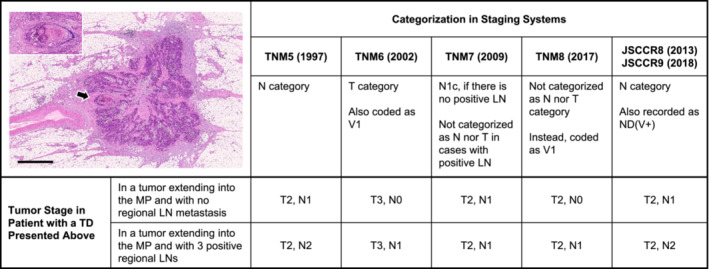
Stage migration caused by different categorizations of a tumor deposit (TD) depending on staging systems. The picture in the upper‐left panel indicates a peritumoral TD with a diameter of approximately 3.5 mm with an irregular contour and an identifiable vascular structure. Under TNM5, this nodule is classified as an LN because it is >3 mm in diameter. On the contrary, this nodule is considered a lesion of the T category because of its contour and is also coded as venous invasion under TNM6. The category N1c is used for this nodule in the absence of regional LN metastasis under TNM7, whereas under TNM8, the tumor stage does not change by this nodule which is regarded as venous invasion because the vascular structure is evident (*arrow*). Since 2013, this nodule has been invariably treated the same as LN metastasis to derive the final N stage in Japan. *Picture*, hematoxylin and eosin staining; *bar*, 1 mm. The inset illustrates the magnification of the part of the nodule that is indicated with an arrow (Victoria blue–hematoxylin and eosin staining). LN, Lymph node; MP, Muscularis propria; TD: Tumor deposit

TD definition has increased in complexity in TNM7, which demands pathologists to distinguish whether the origin of the TD is LNM or not. Furthermore, the lesions with histological evidence of identifiable vascular or neural structure are not regarded as TDs by definition in TNM8. Conceivably, this is based on the concept that TDs should only be applied to lesions having no identifiable origin,^18^ but this is becoming a cause of stage migration (Figure 4). All isolated tumor lesions in the mesocolon or mesorectum, including LNM, have originated from migrated tumor cells that travel via the preexisting anatomical structures, such as the lymphatic or venous systems, or less frequently, the neural system, regardless of whether these are pathologically evident or not. Differentiating the treatment of TDs based on their assumed origin in the absence of clear evidence that such pathological practice benefits patients in planning postoperative treatment would not be logical.

Currently, approximately one in five patients with nodular TDs also show signs of venous or perineural invasion in the nodule, and such lesions have valuable prognostic information.^11^ The estimated 5‐year survival rate of patients with such lesions was as low as 30%–45% based on a single‐center study and two cohorts in the JSCCR's multicenter study.^11^, ^15^ In Japan, since 2013, these nodules have been recorded in pathological reports with the symbols ND(V+), ND(Pn+), or ND(V&Pn+) and are incorporated in tumor staging, similar to other nodular TDs.^19^

### 3.2 What should be the distance of TDs from the primary tumor?

**FIGURE 5:**
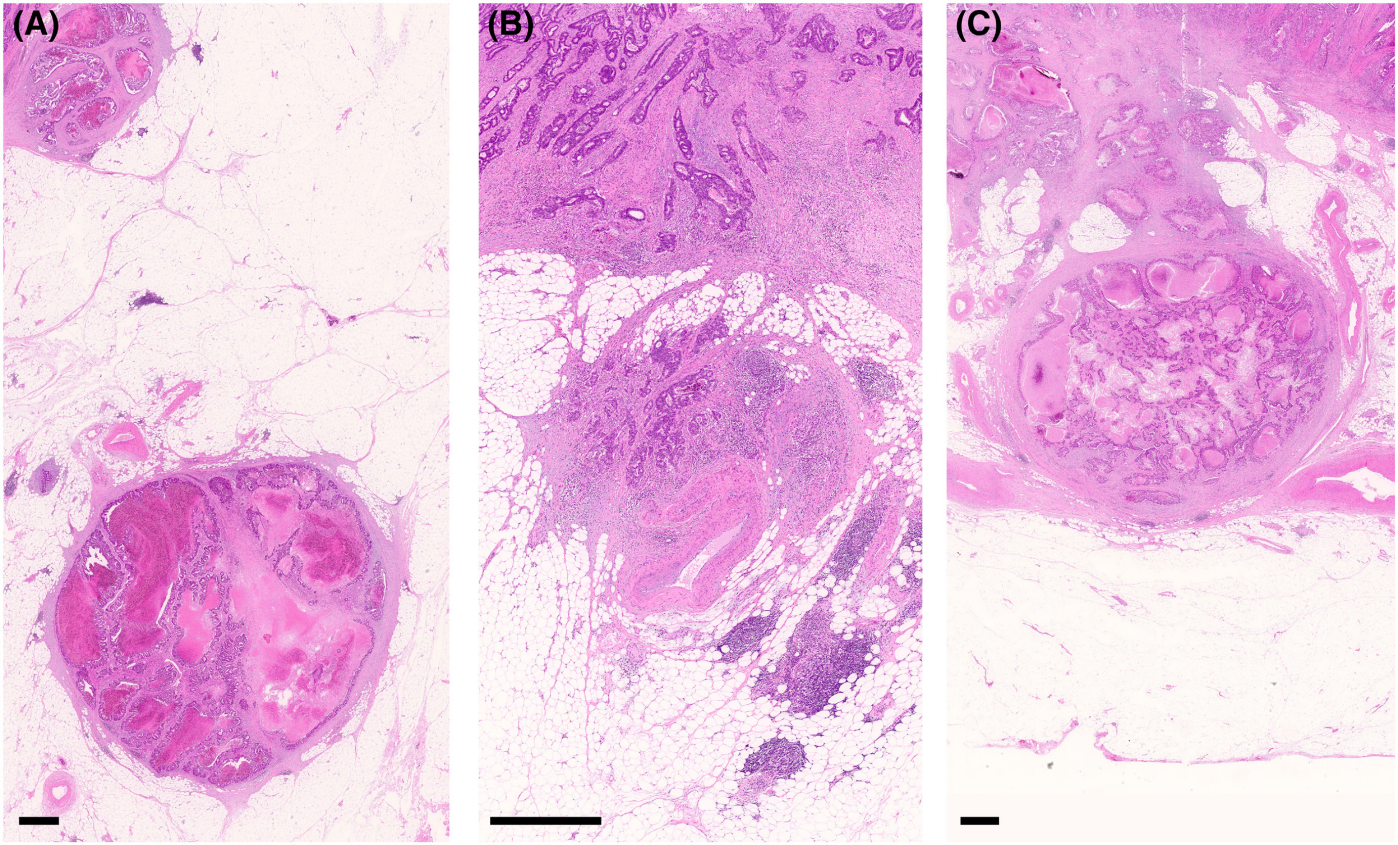
The distance of peritumoral TDs to be located from the body of the primary tumor. The UICC defines tumor deposits (TDs) as discrete macroscopic or microscopic nodules of cancer in the pericolorectal adipose tissue's lymph drainage area of a primary carcinoma that are discontinuous from the primary, but the objective judgment is difficult for the discontinuity. (A) A nodule located at 7.5 mm from the body of the primary tumor; (B) a nodule that is located just below the body of the primary tumor and some streaks of fibrous tissue connecting them; (C) a nodule that is connected to the body of the primary tumor with cancerous tissue. In Japan, among these nodules, only the nodule (A) is regarded as a TD that should be recorded and treated as an N factor according to the “5‐mm” rule for the discontinuity of TDs (Japanese classification of colorectal, Appendiceal, and anal carcinoma, third English edition). (A–C), hematoxylin and eosin staining; *Bar*, 1 mm

Since TNM5, uncertainties were observed in the UICC/AJCC definition concerning the area of peritumoral TD evaluation. Specifically, the distance from the primary tumor or the bowel wall for an isolated tumor lesion to be diagnosed as a TD has no consensus.^16^, ^17^ Approximately 16% of T3/T4 tumors have peritumoral deposits discontinuously located at >2 mm from the body of the primary tumor and the muscularis propria.^20^ TNM8 recommended the lesions to be “discontinuous” from the primary tumor to be classified as TDs, but without specific criteria for judging this “discontinuity,” resulting in a great deal of inconsistency in TD diagnoses (Figure 5). Nagtegaal and Quirke brought up the difficulties that arise when determining whether a TD is a deposit or just a continuous growth of tumor, causing TD misdiagnoses.^21^ Certainly, such a difficulty may well be understood in pathological images of TDs used in some studies, in which the TD is so close to the main body of the primary tumor that it is located within the connective tissue extending directly from the primary tumor.^22^, ^23^, ^24^

Since 2013, guidance for this issue was provided in the Japanese Classification of Colorectal Carcinoma (8th Japanese edition),^25^ wherein all deposits located in the extramural fatty tissue are regarded as TDs in a tumor that is otherwise diagnosed as T1 or T2. A yardstick of 5 mm of discontinuity is used as a criterion for judging a peritumoral TD for T3 or T4 tumors, i.e., only a deposit discontinuously located ≥5 mm from the main body of the primary tumor can be attributed to the final pathological stage (Figure 1). The 5‐mm criterion for peritumoral deposits had been arbitrarily determined based on the consensus of the committee in the JSCCR, who emphasized the results of a multicenter study in which the prognostic value of tumor stage had been improved by the pre‐planned assessment criteria for TDs, including the 5‐mm criterion.^11^, ^14^, ^26^

Similarly, other pathologists attempted to make a yardstick for the term “discontinuously.” For example, Gopal et al. determined that at least 1 cm from the advancing edge was needed to diagnose TDs.^23^ Conversely, Frankel and Jin considered the tumor nodule a TD irrespective of the distance of the tumor nodule from the leading edge of the tumor, when there is no clear connection and the nodule appears discrete.^27^ However, this manner requires demanding costly deeper sectioning to ascertain whether there is truly no connection from the leading edge of the primary tumor body.

## 4 TD CATEGORIZATION IN THE TNM CLASSIFICATION

The TNM classification has categorized TDs based on the “size rule” in TNM5, the “contour rule” in TNM6, and placed them into the “N1c category” in TNM7 and TNM8. More specifically, a tumor nodule of >3 mm is classified as the N category and up to 3 mm as the T category in TNM5. The size rule might be introduced in the presumption that the larger the TD was, the more likely that it originated in LNM, although the rationale for the cut‐off was not disclosed. In TNM6, TDs were classified in the N category if the nodule had the form and smooth contour of an LN.

Size or contour criteria are not presented in TNM7 or TNM8. TDs were no longer treated as a T category,^28^ although there is a confusing description in the 4th (2012)^16^ and 5th editions (2019)^17^ of the TNM Supplement that discontinuous extramural extension becomes a reason for a “pT3” diagnosis if there is regional LNM. TDs were classified as N1c in tumors that would otherwise be classified as N0 in TNM7. The number of TDs does not affect the N category although TDs should be separately counted and recorded in pathological reports.^16^, ^17^ Also in the TNM8, the N1c category is used for all tumors with any T stage, as long as all regional LNs are negative on pathological examination.

### 4.1 Scientific evidence for TNM classification revisions

After the publication of TNM6 that introduced the “contour rule,” by which TDs were classified as LNM or venous invasion depending on whether the contour was smooth or irregular, the weak scientific background in the process of revising the TNM system was criticized.^21^ Already for TNM5 there were issues as the size rule had been established based on a study that was not subsequently published.^29^ Similarly, the contour rule was introduced into TNM6 based on three small studies,^29^ in which only 348,^6^ 344,^7^ and 400 single‐center patients^10^ were analyzed, respectively. None of these studies were intended to assess the prognostic relevance of TD in terms of its shape, and only the prognostic impact of TDs was reported according to their TD criteria. Quirke et al. questioned the validity of the TNM6 criteria for TDs because of the contour rule.^30^

After the revision from the “size rule” to the “contour rule,” we experienced further TNM classification system revisions, but the process is not substantiated by any clear scientific evidence, thereby inviting criticism that TNM should be restructured on a basis equivalent to evidence‐based guidelines.^31^

### 4.2 TD categorization and its relevance to the prognostic value of tumor staging

#### 4.2.1 Advantages and disadvantages of the N1c category

The category of N1c was reportedly created to make a prognostic subgroup for oncologists who were in a quandary about how to treat patients who had TDs but lacked positive LN in terms of adjuvant therapy administration.^32^ Additionally, the name of the category “N1c” was selected because the letter c was the subsequent letter in the alphabet and not necessarily to suggest prognosis.^27^ N1c is repeatedly shown to not indicate poorer survival outcome than N1b according to propensity score matching analyses on the Surveillance, Epidemiology, and End Results (SEER) database.^33^, ^34^

Putting all evidence for the prognostic value of TDs into context, a new category of N1c would be regarded as “partially” successful in terms of prognostic stratification. Specifically, the TNM7 system has successfully achieved its purpose because the evidence is suggesting that N1c identifies a group of patients with poor survival outcomes, thereby indicating the necessity of adjuvant therapy in patients without LNM.^35^, ^36^ Conversely, the current definition of N1c is an obstacle in effectively utilizing the full prognostic information of individual TDs. TDs are only affecting the tumor stage in patients with no positive LNs in the TNM system; furthermore, the number of TD is not considered in deriving the final tumor stage. We can hardly agree that the current TNM system successfully maximizes its performance of prognostic prediction^26^ because of the substantial prognostic information in the number of TDs regardless of LN positivity.^10^, ^11^, ^36^, ^37^

#### 4.2.2 Tumor staging with the “counting” principle

In 2007, a first reported single‐center study aimed to clarify how TDs should be treated in tumor staging and revealed that N staging was capable of more accurately predicting survival outcomes than TNM5 or TNM6 when the number of nodular TD was added to that of positive LN to derive the final pN stage irrespective of the size, contour, or estimated original structure (the “counting” principle).^15^ The validity of the “counting” principle was strongly validated in two multicenter cohorts in a study projected by the JSCCR.^26^ An important result obtained in the JSCCR study was that increasing numbers of nodular TDs were associated with adverse survival outcome. More importantly, statistic indexes for tumor staging were in favor of a revised staging system based on the “counting” principle compared to the TNM7 system in both N and TNM stages.^26^

Recently, the prognostic value of the “counting” principle was validated by an increasing number of studies (Table 3). Song et al. analyzed 513 patients with CRC to compare the alternative staging system based on the “counting” principle to the TNM7 system and revealed the superiority of the “counting” principle in terms of prognostic prediction.^38^ Similarly, the multicenter database of Lie et al. with 4121 patients with stage II and III CRC revealed that the revised pN category based on the “counting” principle was superior to the TNM7 pN category for predicting DFS and OS.^39^ Additionally, two reports from Pei et al. analyzed the SEER database of patients with stage III CRC (21 290 patients treated between 1975 and 2016^40^ and 9198 patients between 2010 and 2016^41^), both of which demonstrated that the “counting” principle improved the prognostic performance of pN and TNM stages.

**TABLE 3:** Scientific literature reporting the value of new staging based on the “counting” principle for categorizing tumor deposits (TDs) in colorectal cancer

Authors (publication year)	Study design	Patients examined	Summary of the results
Ueno et al. (2007)^15^	Retrospective, single‐center study	1027 patients with T2–T4 CRC (1980–1999)	pN^new^ was superior to pN^TNM5/TNM6^ in AIC for CSS, when TDs other than intravascular TD were added to the LNM count.
Ueno et al. (2012)^26^	Retrospective, multicenter study	1716 (1994–1998: first cohort) and 2242 (1999–2003: second cohort) patients with stage I–III CRC	pN^new^ and pTNM^new^ were superior to pN^TNM5/TNM6/TNM7^ and pTNN^TNM7^, respectively, in AIC and C‐index for DSS, when nodular TDs were added to the LNM count.
Song et al. (2012)^38^	Retrospective, single‐center study	513 patients with stage III CRC (1994–2007)	pN^new^ and pTNM^new^ were superior to pN^TNM7^ and pTNM^TNM7^, respectively, in C‐index for CSS, when TDs were added to the LNM count.
Li et al. (2016)^39^	Retrospective, multicenter study	4121 patients with stage II and III CRC (2004–2011)	pN^new^ was superior to pN^TNM7^ in C‐index for DFS and OS, when TDs were added to the LNM count.
Nagtegaal et al. (2017)^9^	Systematic review and meta‐analysis	17 studies comprised 10 106 patients with CRC (1964–2013)	An increasing number of TD was associated with poor outcomes. The combination of TD and LNM was associated with a significantly higher risk of liver metastasis than LNM alone.
Delattre et al. (2020)^42^	Post hoc analysis of a clinical trial	1942 patients with stage III colon cancer (2009–2014) in the IDEA France study	Patients upstaged from pN1 to pN2^new^ by the addition of TD to LNM count had a significantly worse DFS than those with pN1^new^, and it was comparable to pN2.
Pei et al. (2020)^40^	Retrospective database analysis	21 290 patients with stage III CRC from the SEER database (1975 to 2016)	pN^new^ and pTNM^new^ were superior to pN^TNM8^ and pTNM^TNM8^, respectively, in AUC and AIC for OS, when TDs were added to the LNM count.
Pei et al. (2020)^41^	Retrospective database analysis	9198 patients with stage III CRC from the SEER database (2010–2016)	pN^new^ was superior to pN in AUC and AIC for OS, when TDs were added to the LNM count.
Cohen et al. (2021)^43^	Post hoc analysis of a clinical trial	2028 patients with stage III CRC (2010–2015) included in the CALGB/SWOG 80702 study	Patients upstaged from pN1 to pN2^new^ by the addition of TD to LNM count had significantly worse DFS and OS than those with pN1^new^, and they were comparable to pN2.
Pyo et al. (2021)^44^	Retrospective, single‐center study	2446 patients with stage III CRC (2010–2019)	Among patients who completed 6 months of adjuvant chemotherapy, those upstaged from pN1 to pN2^new^ by the addition of TD to LNM count had a significantly worse DFS than those pN1^new^, and it was comparable to pN2.

Abbreviations: AIC, Akaike's information criterion; AUC, area under the receiver‐operating characteristic curve; C‐index, Harrell's concordance index; CRC, colorectal cancer; CSS, cancer‐specific survival; DFS, disease‐free survival; DSS, disease‐specific survival; OS, overall survival; pN^new^ and pTNM^new^, revised pN and pTNM based on the “counting” principle, respectively, i.e., a new method of categorization with adding the number of tumor deposits (TDs) to the number of LNMs to derive a final N stage; pN^TNM5/TNM6/TNM7^, pN according to the definition of TNM5, TNM6, or TNM7; pTNM^TNM7^, pTNM according to the definition of TNM7; SEER, Surveillance, Epidemiology, and End Results.

Two post hoc analyses of a phase III study for stage III colon cancer reported the value of the “counting” principle, the IDEA France study (1942 patients)^42^ and the CALGB/SWAG 80702 study (2028 patients).^43^ The proportion of patients having TD was reported quite differently as 10% in the IDEA France^42^ and 26% in the CALGB/SWAG0702,^43^ presumably due to variation in diagnostic criteria for TDs between France and the United States and Canada. However, the prognostic outcomes of patients who were restaged from pN1 to pN2 by the “counting” principle were similarly shown in two studies, i.e., their DFS rate was significantly lower than that of patients confirmed with pN1 and was comparable to that of patients initially staged as pN2.^42^, ^43^ Additionally, Pyo et al. estimated the prognostic power of modified staging based on the “counting” principle in patients who completed 6 months of CAPOX treatment.^44^ Patients upstaged to N2 from an initial stage of N1 experienced significantly worse 3‐year DFS compared to those remaining in the N1 stage (73% vs. 89%), which was comparable to patients initially staged as N2.

A limited number of studies reported that the concept of the N1c category could be reasonably accepted, ignoring the number of TDs. A study from China concluded that the number of TDs was not a prognostically significant parameter in the TNM staging system because they found no difference in survival outcome between patients having one TD and those with >1 TDs in any examined substage except for T3N1c.^35^ However, the number of patients included in each substage was as small as only 3–42. Some pathologists in the United States have endorsed the current TNM staging system and argued that the number of TDs should not be added to the total number of positive LNs,^27^ but the evidence is lacking for this argument in terms of whether the current TNM system truly achieves the optimal prognostic grouping in patients with CRC.

### 4.3 Diagnostic reproducibility of tumor staging

Under the current UICC/AJCC definition for TNM staging, pathologists have to distinguish a TD from some other isolated tumor lesions, such as (1) an intravascular or perineural deposit, (2) a totally replaced LN, and (3) a tumor nodule accompanied by venous or perineural invasion, to derive the final TNM stage. Three multi‐institutional studies addressed the issue of judgment reproducibility of TDs. The JSCCR study which involved 11 hospitals revealed a 0.74 kappa coefficient for distinguishing LNM from TDs and 0.51 for distinguishing between smooth‐contour nodules as a totally replaced LN and other types of discontinuous lesions.^14^ Similarly, Brouwer et al. reported that the kappa value for the distinction between “nodal” or “non‐nodal” origin was only 0.38 when evaluated by eight experienced gastrointestinal pathologists.^18^ Rock et al. indicated that the complete agreement on the distinction between LNM and TDs was less than half under the definition of the AJCC 7th edition even among pathologists with a specific interest in gastrointestinal pathology.^45^ All these results highlight the difficulties of achieving sufficient interobserver agreement in distinguishing different types of discontinuous cancer spread lesions. Consequently, at present, there is substantial interobserver inconsistency in the tumor staging of individual patients caused by pathological practice for TDs at the moment. The “counting” principle, wherein an individual nodular TD is to be equally treated as positive LNs irrespective of the size, contour, or estimated original structure, would be a promising, one‐size‐fits‐all solution for this challenging situation.

## 5 INTERNATIONAL CONSENSUS NEEDED FOR FUTURE REVISIONS OF TNM CLASSIFICATION

Uncertainty and confusion still remain regarding the role of TDs in tumor staging as listed in Table 2. The definition of peritumoral TDs in terms of the distance from the main tumor is an important issue, and an international consensus on the definition of “discontinuity” for TD is warranted. In Japan, the 5‐mm criterion to define a peritumoral TD is already employed across the country. The wide‐ranging practices found among the literature also highlight the need for international consensus on how to handle pathological specimens, such as the number of sections needed for the diagnosis of TDs, how to count the number of TDs, and whether to use immunohistochemical staining as an adjunct of diagnostic tool. Furthermore, we have to accumulate clinical data for establishing how we treat intravascular or perineural TD, wherein the lesions are less frequently observed than nodular TDs but give a certain degree of prognostic information about the patient.

The UICC/AJCC has generated the definition and categorization of TDs in the TNM staging system in which the origin of the TDs plays a crucial part. However, no scientific evidence was presented for the prognostic value of the origin of TDs. Furthermore, accurate identification of the origin is impossible rather than challenging,^46^ and it is highly concerning that the diagnostic reproducibility of the TNM classification is impaired due to the diagnostic inaccuracy of TDs.^18^, ^30^ Recent evidence suggests a promising solution, which is to incorporate individual nodular TDs in the N staging based on the “counting” principle. Certainly, we recognize the differences between TDs and LNM on some points, such as anatomical distribution, biological aggressiveness of the primary tumor, and prognostic impact^11^; however, we have to bear in mind that the most important role of tumor staging is, after all, accurate prognostic prediction.

In conclusion, the treatment of TDs in tumor staging should be determined in terms of how it will maximize the prognostic value of TNM classification and its reproducibility. All international initiatives to untangle the issues around TDs need to bring us to the optimal TNM staging by which patients would benefit from the most evidence‐based treatment. We should all aim for an evidence‐based, reproducible, and robust TNM staging system and considering our suggestions should improve the current situation.

## CONFLICT OF INTEREST

Hideki Ueno is a current Associate Editor of the *Annals of Gastroenterological Surgery*.
